# Supplementary material for: Transforming specialized mental health practice: Insights from clients’ perspectives on how to support recovery
Source: PLOS Ment Health. 2026 Mar 20;3(3):e0000575. doi: 10.1371/journal.pmen.0000575 (PMC13004367; doi:10.1371/journal.pmen.0000575)
Supplement: Attachment — Submitted filename: Response to reviewers.pdf [file pmen.0000575.s003.pdf]

Dear editor and reviewers,

Thank you very much for your comments on our manuscript *"Transforming specialized mental health practice: insights from clients' perspectives on how to support recovery."* We highly appreciate the useful comments and suggestions and have revised the manuscript accordingly. Below we provide a point-by-point response to the comments and an explanation of the revisions made to the manuscript, including improvements in clarity, organization, and focus.

Thank you for your willingness to assess the revised manuscript. We look forward to hearing from you regarding our response. If there are any further questions, we will be happy to answer them.

Sincerely,

Dr. Karin Lorenz

Dr. Joyce Bierbooms

Prof. dr. Inge Bongers

Journal Requirements:

Figure Resubmissions: While revising your submission, we strongly recommend that you use PLOS's NAAS tool (https://ngplosjournals.pagemajik.ai/artanalysis) to test your figure files. NAAS can convert your figure files to the TIFF file type and meet basic requirements (such as print size, resolution), or provide you with a report on issues that do not meet our requirements and that NAAS cannot fix.
After uploading your figures to PLOS's NAAS tool - https://ngplosjournals.pagemajik.ai/artanalysis, NAAS will process the files provided and display the results in the "Uploaded Files" section of the page as the processing is complete. If the uploaded figures meet our requirements (or NAAS is able to fix the files to meet our requirements), the figure will be marked as "fixed" above. If NAAS is unable to fix the files, a red "failed" label will appear above. When NAAS has confirmed that the figure files meet our requirements, please download the file via the download option, and include these NAAS processed figure files when submitting your revised manuscript.

Response: Thank you for your guidance regarding the figure requirements. The revised manuscript now includes the NAAS-processed figure files to ensure full compliance with the journal's formatting and technical requirements

Reviewers' comments:

Reviewer's Responses to Questions

- Have the authors made all data underlying the findings in their manuscript fully available (please refer to the Data Availability Statement at the start of the manuscript PDF file)?
  Reviewer #2: No

Reviewer #3: No

Response: We understand and appreciate the importance of data transparency. However, in this case, we are unable to make the full interview transcripts publicly available due to ethical and privacy concerns. The transcripts contain detailed personal accounts from clients about their experiences in mental health care with the Open Dialogue approach. Because of the specific content and context, there is a real risk that professionals or others involved in their treatment could recognize them.

Unfortunately, full anonymization cannot be guaranteed, and we feel a strong responsibility to protect the confidentiality and trust of our participants. For that reason, we are not able to share the complete data set publicly.

- Please use the space provided to explain your answers to the questions above. You may also include additional comments for the author, including concerns about dual publication, research ethics, or publication ethics. (Please upload your review as an attachment if it exceeds 20,000 characters)

Reviewer #2: Authors deeply revised the paper according to editorial and reviewers' suggestion; the paper could be accepted in its current revised form

Reviewer #3: See attachment.

Point-by-point response to the comments of reviewer #3
Overall: While the authors have substantially shortened the paper, the need for a tighter focus and better organization remains. A thorough review aiming to remove repetition and ensure content is located under the applicable subheadings is recommended.

Response: We thank you for this suggestion. We have carefully reviewed the manuscript to improve clarity, reduce repetition, and ensure that content is consistently located under the appropriate subheadings. At the same time, we addressed your specific questions (see below), which led to a number of elaborations, by incorporating concrete examples and clarifications, which we believe strengthen the manuscript. Nevertheless, we have aimed to maintain a clear and focused narrative throughout the revised version.

Introduction

1. Consider adding subheadings. For example, before paragraph 4, add subheading “Peer-Supported Open Dialogue.” Before the last paragraph, add subheading “Purpose.”
   Response: We have followed the suggestion and added the proposed subheadings to improve the structure and readability of the text (see Introduction, line 67 and line 99 in the 'Revised Manuscript without Track Changes').

2. Line 68: “explored” or “adopted”?
   Response: We have revised the sentence by adding “and (partly) adopted.” While the approach went beyond mere exploration, it would be inaccurate to describe it as fully adopted. In practice, organizations differed in their level of adoption, and the process was largely characterized by ongoing exploration. The revised wording better reflects this variation (see Introduction, line 68 in the 'Revised Manuscript without Track Changes'):
   “In the Netherlands, several specialized MHC organizations have explored and (partly) adopted Peer-supported Open Dialogue (POD) as a transformative model for recovery-oriented, person-centered network care.”

3. Line 76: “It aims to reject biologically reductionist…”
   Response: We partially agree with the reviewer’s suggestion. Open Dialogue explicitly rejects biologically reductionist views as a core theoretical position. However, the reduction of therapeutic hierarchies is an aim that may not always be fully realized in practice, as it depends on relational and contextual factors. We have revised the sentence accordingly to reflect this distinction (see Introduction, line 76 in the 'Revised Manuscript without Track Changes'):
   It rejects biologically reductionist views, aims to reduce therapeutic hierarchies, and centers clients within relational systems [15, 38].

4. Line 82: This approach seeks to empower…
   Response: We agree with the reviewer and have revised the sentence to “seeks to empower” (see Introduction, line 92 in the 'Revised Manuscript without Track Changes').

5. Line 83: “stories” or “trajectories”?
   Response: We agree with the reviewer and have replaced “stories” with “trajectories” to avoid redundancy with “narratives” later in the sentence and to better capture that empowerment extends beyond ownership of one’s story. (see Introduction, line 92 in the 'Revised Manuscript without Track Changes').

Methods

6. Consider using the following subheadings: Design, Ethics, Setting, Recruitment and consent, Participants, Interview procedures, Data management and analysis, Reflexivity.
   Response: We appreciate this suggestion and have revised the Methods section to follow the proposed subheadings.

7. Revise the “Setting” section to ensure only information describing the setting is included. Remove or move information about the study procedures, design, and participants.
   Response: Thank you for the suggestion. We have revised the “Setting” section (lines 122-152 in the 'Revised Manuscript without Track Changes'), removing unnecessary details and relocating information about study procedures and participants to the appropriate sections to streamline the text and avoid repetition.

8. Given the journal’s international readership, it would be helpful to include information about the local context (socio-economic, culture, etc.).
   Response: We agree that this contextual information is relevant. We have expanded the Methods/Setting section to provide more detail on the Dutch mental healthcare system, relevant cultural factors, the regional characteristics of the Eindhoven - De Kempen area and socioeconomic status (see Setting, lines 123-136 in the 'Revised Manuscript without Track Changes')

9. Line 110: What types of professionals completed the training and formed the POD team?
   Response: The POD team included professionals from a range of disciplines, namely psychiatrists, psychologists, social workers, occupational therapists, and peer-support workers. We have added this information to the Setting section (lines 141-143 in the ‘Revised Manuscript without Track Changes’).

10. Please explain if/how many of the study participants were transitioned from FACT to POD and if/how many of the study participants were new clients assigned to POD.
    Response: All participants were transitioned from a FACT team to the POD team; no participants were newly assigned to POD. We have added this information to the Methods section (see Participants, lines 171-172 in the ‘Revised Manuscript without Track Changes’).

11. For how long did participants engage in POD care before the interviews?

Response: Prior to participating in the study, six participants had attended more than 50 network meetings, four participants had attended more than 30, and six participants had attended more than 20 network meetings. We have added this information to the Methods section (see Participants, lines 181-183 in the 'Revised Manuscript without Track Changes').

12. Were participants compensated or incentivized?
    Response: No, participants were not compensated or provided with incentives for taking part in the study. Participation was entirely voluntary, and participants were informed of this beforehand. This information has been added to the Ethics section (lines 120-121).

13. Line 160: please review names of diagnoses to ensure congruency with a diagnostic system. For example, trauma is not typically referred to as a diagnosis. Would the authors be referring to post-traumatic stress disorder? Please clarify.
    Response: We have reviewed the terminology used for diagnoses; however, the terms reported in the manuscript reflect the words that participants themselves used to describe their difficulties. In line with the principles of POD, we aimed to remain close to participants' own language and understanding of their experiences, rather than imposing formal diagnostic labels. We did not cross-check the participants' electronic health records to verify formal diagnoses in the medical system. We have clarified in the text that this terminology comes directly from the participants (see Participants, lines 184-185 in the 'Revised Manuscript without Track Changes'):

    "Mental health conditions, as reported by participants themselves, included psychosis, trauma, anxiety, depression, bipolar disorder, and addiction."

14. Line 162: What questions / prompts were used? Consider including the interview protocol as an appendix.
    Response: We thank the reviewer for this comment. Interviews were guided by a semi-structured topic list rather than a fixed question protocol, allowing participants to elaborate on issues most relevant to their experiences. The topic list was used flexibly, enabling participants to focus on the aspects of POD care that were most meaningful to them, while also addressing core themes such as involvement, support, network meetings, and recovery. To increase transparency, we have included representative example questions in the manuscript (see Interview procedures, lines 192-195), including both positive and critical prompts (e.g., "How did you experience the support from the POD team in your recovery process?"; "Were there aspects that did not

contribute to your recovery?"). We hope that this clarification and the inclusion of example questions enhance transparency regarding the interview approach.

15. Some of the content deleted in the first round of revisions should be reintegrated:
    - Line 166: Please clarify what 'on-site' refers to. In a private office at the organization's building?
    - Bring back "interviews were audio-recorded."

    Response: This information has been reintroduced in the revised manuscript (see Interview procedures, line 188; and Data management and analysis, line 201 in the 'Revised Manuscript without Track Changes')

16. I'm assuming the interviews were conducted in Dutch. Please add a statement explaining at what point data were translated and by whom.

    Response: The interviews were conducted in Dutch, and all analyses were performed on the original Dutch transcripts. The results were subsequently translated into English, with co-author review to ensure conceptual accuracy. We have clarified this in the manuscript by adding a statement to the Interview procedures section (line 187):
    "Interviews were conducted in Dutch and lasted 1–1.5 hours."

    In addition, we have specified the timing and process of translation in the Data management and analysis section (lines 211-213):
    "Themes were reviewed, refined, and defined. Analyses were conducted on the original Dutch transcripts, and the results were subsequently translated into English with co-author review to ensure conceptual accuracy."

17. Please explain what exactly project conversations are when they are first mentioned.

    Response: We have clarified that interviews constituted the primary data collection for this study. The project-based conversations are now described as existing qualitative material collected prior to the study period and conducted using the same semi-structured format and topic list to ensure comparability across data sources (See Design, lines 108-112 in the 'Revised Manuscript without Track Changes'):
    "This qualitative, practice-oriented field study was conducted from November 2018 to February 2021 within a broader POD project that established a multidisciplinary outpatient POD team (see Setting). Primary data collection for the present study consisted of semi-structured in-depth interviews exploring clients' experiences with POD care. In addition, existing qualitative material from project-based semi-structured conversations was included. These conversations had been conducted prior to the study period as part of routine quality improvement activities within the POD project and

followed the same semi-structured format and topic list as the research interviews. Including this material allowed us to enrich the dataset while maintaining methodological consistency across data sources."

18. Please justify the choice of using both individual interviews and project conversations.
    Response: We used both individual interviews and project-based conversations to enrich the empirical basis of this practice-oriented qualitative study. The project-based conversations contained in-depth experiential data that closely aligned with the study's research aim and were conducted using the same semi-structured format and topic list as the research interviews. Including this existing material allowed us to maximize data richness while maintaining methodological consistency. All project-based conversations had been conducted with informed consent for scientific use and were analyzed using the same analytic procedure as the interviews. We have clarified this rationale in the Design section (lines 112-114, in the 'Revised Manuscript without Track Changes') as follows:
    "*Primary data collection for the present study consisted of semi-structured in-depth interviews exploring clients' experiences with POD care. In addition, existing qualitative material from project-based semi-structured conversations was included. These conversations had been conducted prior to the study period as part of routine quality improvement activities within the POD project and followed the same semi-structured format and topic list as the research interviews. Including this material allowed us to enrich the dataset while maintaining methodological consistency across data sources.*"

19. Line 187: Remove "CHIME" as it is not mentioned or defined anywhere else in the paper.
    Response: We have removed the reference to CHIME (in line 190, in the 'Revised Manuscript without Track Changes') from the manuscript.

20. Line 189: Did the POD team confirm the validity of the preliminary findings?
    Response: Preliminary findings were shared with the POD team to validate interpretive credibility. The team recognized the findings as consistent with their professional experience with the clients, confirming that the analyses accurately reflected participants' perspectives and providing no additional comments. This clarification has been added to the end of the Reflexivity section (lines 224-226) of the revised manuscript:
    "The POD team recognized the preliminary findings as consistent with their experience with the clients, confirming that the analyses accurately reflected participants' perspectives and providing no additional comments."

Results

Overall, results need improvement in terms of coherence and flow. The quotes don't always exemplify the subtheme and the wording needs to be more carefully chosen for increased clarity. Additionally, the quotes need to be better integrated, interwoven in the narrative for better flow. Lastly, including more concrete examples of how the various processes took place would substantially strengthen this paper, as it all sounds somewhat abstract as is. Below I highlight a few specific examples, but this section should be thoroughly reviewed for these patterns.
Response: We acknowledge that the Results section could be improved and it has now been thoroughly revised to enhance coherence, narrative flow, and clarity (See Results section of the revised manuscript). Quotes are carefully integrated to illustrate each subtheme and are supported by concrete examples of processes, such as reflection moments, network meetings, and professionals' responsiveness in practice. While individual quotes cannot capture the full complexity of participants' experiences, they were selected to exemplify key patterns and insights, supporting rather than replacing the analytic narrative. These revisions aim to address the concerns regarding clarity, flow, quote integration, and concreteness.

21. The first paragraph should list the overarching themes interpreted from the data analysis, in this case, I believe it would be the three building blocks. Consider beginning the section with the content currently starting in line 204.
    Response: We have revised the opening of the Results section to begin with the overarching themes derived from the data analysis. The section now opens by explicitly introducing the three interrelated building blocks that emerged as central to POD's contribution to clients' recovery, followed by a brief synthesis of participants' overall experiences. The Results section has been reorganized accordingly, and the content previously starting at line 204 has been moved to the beginning of the section (see revised Results section, lines 228-248).

22. Line 218: "Participants reflected on/described / highlighted…how POD professionals emphasized strengths and offered positive reinforcement." Revise results section thoroughly for this type of structuring.
    Response: We have thoroughly revised the Results section to ensure consistent structuring, clearly linking participant reflections.

23. Table 2. Consider adding a third column to include a quote for each of the subthemes.

Response: We appreciate the reviewer's suggestion to add illustrative quotes to Table 2. We carefully considered this recommendation. However, the primary purpose of Table 2 is to provide a concise overview of the main building blocks and subthemes, as requested by previous reviewers, offering readers a structured summary of the analytic findings.
Including quotes for each subtheme would substantially expand the table and risk shifting its function from an analytical overview to an illustrative presentation of data. As individual quotes capture only specific aspects of each subtheme, we believe they are more appropriately integrated within the narrative Results section, where they can be contextualized and analytically interpreted.
We have therefore retained the current structure of Table 2, while thoroughly revising the Results section to ensure that each subtheme is clearly supported by carefully integrated quotes and concrete examples. We hope this approach maintains both clarity and analytical coherence.

24. Line 243: What does "Aligning with one's own story" mean? Who is "one" referring to? The text indicates a meaning related to engagement with the story. Please provide a clearer definition or explanation of this subtheme.
Response: We agree that the original formulation of "Aligning with one's own story" could be interpreted as ambiguous. In the revised manuscript, we have clarified that "one" refers to the client and that this subtheme captures how POD professionals attune themselves to clients' personal narratives, supporting them in remaining authors of their own stories rather than imposing professional interpretations. Specifically, we now make explicit that alignment refers to preserving clients' interpretive authority over their experiences and creating space for them to articulate and make meaning of their difficulties in their own terms. This clarification has been added at the beginning of section 3.1.3 (lines 287-288) to make the analytic meaning of the subtheme more explicit.

25. Line 259: Similarly, what is "tolerance" referring to? Do the authors mean "self-determination"?
Response: The term "tolerance" may indeed have been ambiguous. By "tolerance," we refer to professionals deliberately allowing space for clients' self-direction, refraining from taking over decisions, and accepting uncertainty or non-linear progress within the recovery process. In the revised manuscript, we have clarified this by explicitly describing how professionals created space for initiative and personal responsibility, while also emphasizing the need for guidance at vulnerable moments.

Thus, “tolerance” does not refer to self-determination itself, but to the professionals’ stance of allowing clients’ autonomy while remaining available for attuned guidance. This clarification has been incorporated in section 3.1.4 (lines 317-319).

26. Line 269: Consider using “human connection” instead of “human-to-human connectedness”

    Response: We thank the reviewer for this suggestion. We agree that “human connection” is stylistically clearer and more idiomatic in English. In the revised manuscript, we have therefore replaced “human-to-human connectedness” with “human connection” throughout the manuscript to ensure consistency.

    To preserve our intended meaning, we clarified in the text (lines 333-335) that this subtheme refers to a reciprocal, person-to-person mode of relating, in which both professionals and clients remain present as human beings rather than solely as role-bearers.

27. Line 288: This is an important point. While there may be an effort to bridge the power differential between provider and client, it is very important to acknowledge that there will always be some level of power differential / hierarchy in the professional / client relationship. Consider expanding on this in the discussion.

    Response: We appreciate the reviewer’s comment regarding the power differential in professional - client relationships. Upon reflection, we concluded that the original subtheme title, “A non-hierarchical personal connection,” could give a misleading impression of the absence of structural asymmetry or professional authority. Participants’ experiences indicated that while the relationship felt more personal and reciprocal, professionals remained paid employees with formal responsibilities, boundaries, and institutional authority. Therefore, we have revised the subtheme title to “A more egalitarian personal connection” to better capture participants’ perspectives (line 347).

    In addition, we have added the following text to the Discussion section (lines 524-531) to elaborate on this point:

    “While clients in this study experienced their relationships with professionals as more personal and egalitarian, they also recognized enduring structural asymmetries: professionals remained paid employees with formal responsibilities and institutional authority. The OD approach has been described as challenging established norms and power structures through a paradigm shift [69, 70, 71]. Our findings suggest, however, that this does not imply the disappearance of hierarchy, which aligns with the OD perspective [72]. Rather than eliminating hierarchy, POD appears to recalibrate it,

shifting from directive control toward responsive guidance within an inherently asymmetrical relationship."

28. Line 307: Please explain "reflection moments" earlier in the paper (maybe in the background in the description of POD) as they will be unfamiliar to many readers.
    Response: We have added a clarification to the Introduction section (lines 80-84) of the revised manuscript:
    "Central to the approach are facilitated network sessions, led by at least two professionals trained in OD. *During these sessions, professionals may engage in so-called "reflection moments," in which they briefly share their observations, thoughts, or emotional responses with each other in the presence of the client and their network. These reflections are offered transparently and tentatively, fostering dialogue rather than imposing interpretations* [41, 42]."

29. Line 324: This subtheme seems to be very similar to the "balancing tolerance and need for guidance." Consider combining.
    Response: We understand that the subthemes may appear overlapping at first glance. However, they emphasize distinct aspects of participants' experiences. "Collaboration on an equal footing" highlights the relational dimension of POD care, focusing on equality, mutual respect, and shared decision-making in the recovery process (e.g., a participant described professionals as "not hand in hand, but side by side," emphasizing partnership in recovery [Participant 12]). In contrast, "Balancing tolerance and the need for guidance" emphasizes the procedural and practical aspect of professional support, how professionals calibrate the degree of direction to participants' readiness, timing, and informational needs (e.g., one participant described feeling overwhelmed when a professional expressed uncertainty, highlighting the need for careful attunement [Participant 4]). We have retained both subthemes in the revised manuscript to preserve these analytically distinct insights.

30. Line 350: What are "calm-paced" meetings?
    Response: We clarified that calm-paced refers to meetings proceeding at a relaxed, unhurried rhythm, allowing space for emotional expression, reflection, and deep processing. This explanation has been added in section 3.3.2, lines 436-437:
    "Participants described meetings as calm-paced, meaning that conversations proceeded at a relaxed, unhurried rhythm, providing space for intense emotions, reflection, and deep processing."

31. Line 352: “the absence of time pressure” - this needs to be expanded in the discussion. What structural supports were in place that allowed providers to spend unlimited time and be constantly available for patients? This would not be realistic in most settings.
Response: We believe that we were not sufficiently clear in the Results section: “the absence of time pressure” refers to participants’ perception of having sufficient time to express themselves, process emotions, and engage in reflection, rather than literally unlimited time. In practice, sessions were typically capped (up to 1.5 hours), with professionals balancing responsiveness and practical constraints. To avoid misinterpretation, we now describe it as follows in the Results section (lines 436-440):

“Participants described meetings as calm-paced, meaning that conversations proceeded at a relaxed, unhurried rhythm, providing space for intense emotions, reflection, and deep processing. When professionals extended sessions to accommodate participants’ needs, as one participant noted, ‘They extended the session when they saw I needed more time’ (Participant 15), this was experienced as attentiveness and contributed to feeling genuinely heard.”

Regarding the comment on constant availability, we agree that literal continuous availability would not be realistic and is also undesirable within community-based care. We address this issue in the Discussion section (lines 532-542), noting that clients valued the consistent presence of POD-trained professionals for crises and emotional support, while also discussing the practical and normative tensions of continuous availability in community-based care.

Discussion

32. In addition to the observations made above, consider addressing (briefly) how the socio-economic and cultural context (local, national) may have impacted the program / study / findings.
Response: We thank the reviewer for this suggestion. In the revised manuscript (Discussion, lines 543-548), we briefly highlight aspects of the Dutch socio-cultural context that may have shaped clients’ experiences of POD. The Netherlands is characterized by relatively low power distance and a strong tradition of consultation and shared decision-making, which plausibly facilitated acceptance of dialogical and collaborative practices. We did not specifically examine socio-economic status or cultural influences in this study, and participants’ neighborhoods were approximately average in SES. Consequently, transferability of the findings to settings with different healthcare structures, resources, or professional hierarchies should be considered cautiously.

"Taken together, these findings illustrate how recovery-oriented, dialogical principles are enacted in practice, while highlighting tensions that warrant reflection. They should be interpreted in light of the Dutch socio-cultural and healthcare context, which is characterized by low power distance, a tradition of consultation, and individual autonomy [48]. These conditions may have facilitated clients' acceptance of collaborative practices within POD, so caution is warranted when considering transferability to settings with different structures or professional hierarchies."

33. Another question that lingers is how POD compares to care as usual. Some minor additions (both in the background and discussion) highlighting differences (concrete differences, not theoretical) would provide greater understanding of the extent to which these principles (recovery-orientation, person-centeredness) are better applied through POD.
    Response: We appreciate the reviewer's suggestion to clarify how POD differs from care as usual through concrete examples. We have therefore added explicit descriptions of key practice-level differences in both the Introduction and the Discussion.

    The following clarification has been added to the Introduction, lines 84-90:
    "Central to the approach are facilitated network sessions, led by at least two professionals trained in OD, a format that differs from conventional individual consultations. During these sessions, professionals may engage in "reflection moments," in which they briefly share their observations, thoughts, or emotional responses with each other in the presence of the client and their network. These reflections are offered transparently and tentatively, fostering dialogue rather than imposing interpretations. [41, 42]. In line with the principle "nothing about me without me," no separate case discussions take place without the client present; decisions and clinical deliberations occur within the network meeting itself. OD does not preclude any form of treatment from the outset, nor does it operate with a predetermined agenda or predefined treatment goals, unlike care pathways organized around diagnosis-driven treatment planning. Instead, professionals follow the network's lead through open-ended questions and responsive engagement, allowing concerns, meanings, and potential directions to emerge within the dialogue. Any (evidence-based) intervention can be integrated when the network identifies it as helpful, based on shared understanding and collaborative decision-making. This approach seeks to empower individuals to take ownership of their own trajectories and actively shape their personal narratives [42]."

In addition, the Discussion section (lines 496-497) now includes further specification of how professionals' use of reflection moments and personal transparency distinguishes POD from more conventional forms of care:

"Network meetings with reflection moments involving at least two POD professionals were seen as particularly innovative. In these meetings, professionals openly shared personal impressions and emotional responses, rather than maintaining the more neutral stance typical of conventional care. Conducted with dialogism, tolerance of uncertainty, and reflective practice [41, 42, 56, 57], they created "free spaces" for clients to pursue meaningful, autonomous lives [34]. "

34. Lastly, please address if/how patients are transitioned out of care in this model. This may carry important implications given the deep relational bonds developed with providers.
Response: We acknowledge the reviewer's point regarding transitions or completion of care. While participants' experiences of personal bonds with professionals may influence care trajectories, our interviews did not specifically explore how or when clients are discharged or transitioned. Therefore, this topic is outside the scope of the current article and is not discussed in the Discussion. To provide context, we have added a brief description to the Methods section, Setting (lines 150-152):
"The team serves approximately 285 clients, with 20–30 new referrals annually, and operates in accordance with the Open Dialogue approach, as outlined in the Introduction and summarized in Table 1. Previous studies have described the implementation of POD within this team and its associated challenges [17, 45]. The team provides a continuous, person-centered treatment pathway, covering the clinical process from initial contact to completion of care. Network meetings form the core of the approach, with additional interventions and support offered flexibly in response to clients' needs and preferences. Client transitions from POD follow standard mental health practice: when network discussions indicate that professional support is no longer needed, POD staff gradually step back at a pace tailored to the client's needs."
