# Supplementary material for: Transforming specialized mental health practice: Insights from clients’ perspectives on how to support recovery
Source: PLOS Ment Health. 2026 Mar 20;3(3):e0000575. doi: 10.1371/journal.pmen.0000575 (PMC13004367; doi:10.1371/journal.pmen.0000575)
Supplement: Attachment — Submitted filename: Response to Reviewers.docx [file pmen.0000575.s002.docx]

Dear editor and reviewers,

Thank you very much for your comments on our manuscript *“Transforming specialized mental health practice: insights from clients’ perspectives on how to support recovery."* We highly appreciate the useful comments and suggestions and have revised the manuscript accordingly. Below we provide a point-by-point response to the comments and an explanation on the revisions made to the manuscript.

We would also like to clarify that content of this manuscript has been included as a chapter in the doctoral dissertation of the first author, which is available through the university repository. The dissertation is not commercially published and does not have an ISBN. The version in the dissertation reflects the manuscript as it was originally submitted to *PLOS Mental Health* on March 3, 2025. We trust this will not pose any issues for the review and potential publication process, but we wanted to be transparent about this in case clarification was needed.

Thank you for your willingness to assess the revised manuscript. We look forward to hearing from you regarding our response. If there are any further questions, we will be happy to answer them.

Sincerely,

Dr. Karin Lorenz

Dr. Joyce Bierbooms

Prof. dr. Inge Bongers

**Journal Requirements:**

1. Please describe in your methods section how capacity to provide consent was determined for the participants in this study. Please also state whether your ethics committee or IRB approved this consent procedure. If you did not assess capacity to consent please briefly outline why this was not necessary in this case.

Response: Thank you for this suggestion. Capacity to provide consent was assessed by the principal clinicians of the studied setting, who made a professional judgment on whether eligible clients were able to provide informed consent. The ability to provide informed consent was a prerequisite for participation; clients deemed unable to consent were not approached. This procedure was reviewed and approved by the ethics committee, and we have now explicitly stated this in the Methods section of the manuscript (see Participations and recruitment, lines 146-149 in the Revised Manuscript without Track Changes):

*“During the POD project, clients receiving or entering care could apply to participate. Recruitment occurred in two phases (Nov 2018–Mar 2019; Sep 2020–Feb 2021), with treatment teams actively involved. Eligible participants were adults with SMI, experiencing significant social and societal limitations for at least one year, and who may not have benefited from traditional mental health services [44]. Inclusion was encouraged regardless of recovery stage, treatment motivation, their willingness to seek help, or attitude toward POD—positive, negative, or neutral. Clients with intellectual disabilities, memory impairments, or in psychological crisis were excluded. Capacity to provide informed consent was assessed by the principal clinicians, who made a professional judgment on client’s ability to consent; only those deemed capable were approached, in accordance with the consent procedure approved by the ethics committee.”*

2. We ask that a manuscript source file is provided at Revision. Please upload your manuscript file as a .doc, .docx, .rtf or .tex.

**Additional Editor Comments (if provided):**

1. The paper needs a MASSIVE reduction in length, from over 10,000 words to not more than 5,000, possibly to 4,000 words. The text has to be improved in terms of clarity and results have to be tabulated and shown in tables and figures, with a clear, but concise description of results and a discussion.

Response: Thank you for this valuable suggestion. We have substantially reduced the manuscript, cutting its length by roughly half through streamlining the text and removing non-essential details. The original article was written by the authors, and AI assistance was used solely to help shorten and refine the wording of this revised version. Would you advise that we include a statement about this AI-assisted editing in the manuscript (for example in the Author Contributions or Acknowledgments section), or is it not necessary?

As suggested, we have summarized the key results in a single table for clarity and removed the appendix to prevent repetition of the OD approach, referring instead to the relevant literature. Given the substantial reduction in length, we wonder whether the results table (line 210 in the Revised Manuscript without Track Changes) is still necessary. A further reduction would be possible if this table were removed, and we are happy to follow the editor’s preference on whether to keep or omit it.

**Reviewers' comments:**

**Reviewer's Responses to Questions**

**Comments to the Author**

1. Does this manuscript meet PLOS Mental Health’s [publication criteria](https://eur03.safelinks.protection.outlook.com/?url=https%3A%2F%2Ftrack.editorialmanager.com%2FCL0%2Fhttps%3A%252F%252Fjournals.plos.org%252Fmentalhealth%252Fs%252Fcriteria-for-publication%2F1%2F010f01969267629d-b6428086-9953-4447-933b-a7ff0b659b29-000000%2FqHpJnPUOrlO_L0etWLSfmRCLuSi9g0VsdjIZNNskgEM%3D209&data=05%7C02%7Ckarin.lorenz%40ggze.nl%7Cfe87afcc1c6747e2ea4a08dd9d2f4859%7C8e63ae1811a44bccac6a45f9a20e84ca%7C0%7C0%7C638839548595695629%7CUnknown%7CTWFpbGZsb3d8eyJFbXB0eU1hcGkiOnRydWUsIlYiOiIwLjAuMDAwMCIsIlAiOiJXaW4zMiIsIkFOIjoiTWFpbCIsIldUIjoyfQ%3D%3D%7C0%7C%7C%7C&sdata=aSWdxUMqEGKoV%2BWppfe2dwRFWwRnszVt%2BytImHu6mJE%3D&reserved=0)? Is the manuscript technically sound, and do the data support the conclusions? The manuscript must describe methodologically and ethically rigorous research with conclusions that are appropriately drawn based on the data presented.

Reviewer #1: Yes

Reviewer #2: Partly

2. Has the statistical analysis been performed appropriately and rigorously?

Reviewer #1: Yes

Reviewer #2: N/A

3. Have the authors made all data underlying the findings in their manuscript fully available (please refer to the Data Availability Statement at the start of the manuscript PDF file)?
   The [PLOS Data policy](https://eur03.safelinks.protection.outlook.com/?url=https%3A%2F%2Ftrack.editorialmanager.com%2FCL0%2Fhttps%3A%252F%252Fjournals.plos.org%252Fmentalhealth%252Fs%252Fdata-availability%2F1%2F010f01969267629d-b6428086-9953-4447-933b-a7ff0b659b29-000000%2FI49vydVQuKJdHiUKqbml_pz3JySTfoT9a_F_0a2ysPA%3D209&data=05%7C02%7Ckarin.lorenz%40ggze.nl%7Cfe87afcc1c6747e2ea4a08dd9d2f4859%7C8e63ae1811a44bccac6a45f9a20e84ca%7C0%7C0%7C638839548595709911%7CUnknown%7CTWFpbGZsb3d8eyJFbXB0eU1hcGkiOnRydWUsIlYiOiIwLjAuMDAwMCIsIlAiOiJXaW4zMiIsIkFOIjoiTWFpbCIsIldUIjoyfQ%3D%3D%7C0%7C%7C%7C&sdata=%2FRBYqicq4gCWsBAAxT4zMPlLCF2cXfqXcgjcagBDV7k%3D&reserved=0) requires authors to make all data underlying the findings described in their manuscript fully available without restriction, with rare exception. The data should be provided as part of the manuscript or its supporting information, or deposited to a public repository. For example, in addition to summary statistics, the data points behind means, medians and variance measures should be available. If there are restrictions on publicly sharing data—e.g. participant privacy or use of data from a third party—those must be specified.

Reviewer #1: No

Reviewer #2: No

Response: We understand and appreciate the importance of data transparency. However, in this case, we are unable to make the full interview transcripts publicly available due to ethical and privacy concerns. The transcripts contain detailed personal accounts from clients about their experiences in mental health care with the Open Dialogue approach. Because of the specific content and context, there is a real risk that professionals or others involved in their treatment could recognize them.

Unfortunately, full anonymization cannot be guaranteed, and we feel a strong responsibility to protect the confidentiality and trust of our participants. For that reason, we are not able to share the complete data set publicly. Researchers who are interested in the data are welcome to contact us to discuss possibilities for limited and ethically responsible access.

4. Is the manuscript presented in an intelligible fashion and written in standard English?
   PLOS Mental Health does not copyedit accepted manuscripts, so the language in submitted articles must be clear, correct, and unambiguous. Any typographical or grammatical errors should be corrected at revision, so please note any specific errors here.

Reviewer #1: Yes

Reviewer #2: Yes

**Review Comments to the Author**

**Reviewer #1: Manuscript Review**
The present paper seeks to deepen the understanding of clients’ lived experiences with POD to inform mental health care practices that support recovery. While the manuscript addresses an important and underexplored topic, significant revisions are needed to enhance clarity and completeness before publication.

**General Considerations**

1. Although PLOS Mental Health does not impose word limits, it encourages conciseness. At approximately 10,900 words (excluding references), the manuscript resembles a thesis rather than a journal article, making it challenging to read. I recommend reducing the length to a maximum of 6,000 words—ideally around 4,000. Consider summarizing some results in tables and moving non-essential comments to an appendix. Avoid using the appendix to reiterate a model that has already been thoroughly explained in the introduction; instead, refer to existing literature where appropriate. Overall, unnecessary details should be removed from every section.

Response: We appreciate the reviewer’s feedback on enhancing clarity and brevity. In response, we have significantly shortened the manuscript, reducing its length by about half, by streamlining the text and removing non-essential details. Key results are now summarized in a single table (table 2, line 210 in the 'Revised Manuscript without Track Changes'). In addition, we removed the appendix entirely to avoid any repetition of the OD approach and refer to the relevant literature where appropriate and added a brief description of the OD approach in Table 1 (see Introduction, line 85 in the 'Revised Manuscript without Track Changes'), in line with recommendations of reviewer 2.

Introduction:

2. The transition in lines 49–50 lacks geographical context. Is this change occurring worldwide, in Europe, in Western countries, or exclusively in the Netherlands? Please clarify.

Response: We have revised the sentence to clarify the global scope (see Introduction, line 49 in the 'Revised Manuscript without Track Changes'):

*“Mental health care (MHC) is transitioning globally toward community-based, recovery-oriented, person-centered services [1–8].”*

3. Strengthen the discussion on the importance of clients' identity by citing:
   -DOI: 10.1080/09638237.2024.2426980
   -http://www.bu.edu/cpr/repository/articles/pdf/deegan1996.pdf

Explicitly state that the focus is on personal recovery, distinguishing it from clinical or functional recovery.

Response: Thank you for this valuable suggestion. We have consulted the recommended references and revised the manuscript accordingly (see Introduction, lines 56, 57, 59, 61 and 62 in the Revised Manuscript without Track Changes):

*“This transformation entails a shift in values: recognizing individuals with severe mental illness (SMI) as whole persons, not merely clients [18–20], and enabling them to direct their own lives rather than being passive recipients of care [21]. It calls for a holistic approach beyond the biopsychosocial model [5, 19], viewing recovery as a personal, evolving process that restores agency and meaning [14, 22]. Rather than focusing solely on diagnosis and outcomes (clinical recovery), professionals must help clients pursue lives aligned with their own aspirations [9, 23], empowering both them and their social networks [2, 24–26]. The focus is on personal recovery, which aims not at normalization, but at enabling each individual to flourish as the unique human being they are [27].”*

Methods

4. Remove unnecessary details to streamline this section.

Response: Thank you for this suggestion. We have removed unnecessary details and streamlined the section accordingly.

Results

5. When referring to "most" patients, specify the exact percentage where possible to enhance scientific rigor.

Response: We understand this suggestion and would like to provide additional clarity regarding our approach. Specifically, we followed a phenomenologically informed approach, involving detailed deductive–inductive exploration and iterative hermeneutic interpretation of participants’ experiences. Given that this focus on latent themes goes beyond participants’ explicit statements, we consider quantification through exact percentages inappropriate. Had this been a descriptive qualitative study focusing only on semantic content, reporting percentages might have been more appropriate. To increase transparency and rigor, we have added a brief explanation of this analytic approach to the Methods section (see lines 176-177 and 184 in the Revised Manuscript without Track Changes):

*“All interviews and project-based conversations were transcribed verbatim and analyzed using thematic coding in Atlas.ti [46], following Braun and Clarke’s six-phase framework [46] through an iterative, hybrid approach [47]. Following a phenomenologically informed approach [48], focusing on participants’ experiences, initial codes were generated using both deductive and inductive techniques, informed by POD principles, recovery terminology, and study objectives (e.g., reflection moments, responses to problems, tolerance of uncertainty, team meetings, network participation, connectedness, client narratives, support, challenges, and prerequisites). New codes were added as needed.*

*Codes were then clustered into latent subthemes through reflective iteration. These subthemes were organized under broader themes that captured underlying assumptions and conceptualizations beyond explicit statements [51], reflecting an interpretive, hermeneutic approach [52]. Themes were reviewed, refined, and defined.”*

6. Summarize key results in tables to improve readability and transform the manuscript into a more accessible paper.

Response: Thank you for this suggestion. We have now summarized the requested information in a table in the Results section (see Table 2: Overview of the three building blocks, their subthemes, and concise descriptions. Line 210 in the Revised Manuscript without Track Changes).

Discussion

7. A significant limitation of the study is the limited generalizability due to the small sample size. How many patients typically attend the mental health center (MHC)? Given that your study includes only a small group, provide context on its representativeness.

Response: We recognize the concern about generalizability. However, because this is a qualitative study, our aim was not statistical generalization but to provide transferable insights drawn from rich, detailed accounts of the lived experiences of people with severe and persistent mental illness, a group that is difficult to reach and underrepresented in the literature.

That said, we fully acknowledge that the study included a small sample. We had intended to recruit a larger and more diverse group, including clients less inclined to participate, and repeatedly encouraged the POD team to support broad recruitment. Ultimately, however, participation depended on clients’ and relatives’ willingness and ability to take part in the study.

The participating MHC team has a caseload of about 285 clients, as described in the manuscript (See setting, line 127 in the Revised Manuscript without Track Changes). In total, data from 16 clients (9 male, 7 female) and two female relatives were analyzed. We do not know exactly how many clients were ineligible (e.g., due to intellectual disability, memory impairment, acute psychological crisis, or inability to provide informed consent). Although this represents a small proportion of the caseload, we consider it representative for the caseload, as the group was heterogeneous, encompassing a range of diagnoses and a variety of attitudes toward POD (positive, neutral, and critical). We recognize that individuals who chose to participate may differ from those who declined; people less inclined to share their views might hold different perspectives. We have revised the manuscript accordingly (see Strengths and Limitations, lines 445-454 in the Revised Manuscript without Track Changes):

*“However, limitations must be acknowledged. Recruitment was mediated by POD professionals, potentially introducing selection bias despite efforts to ensure diversity. The participating POD team has a caseload of about 285 clients, and data from 16 clients (9 male, 7 female) and two female relatives were analyzed. The exact number of ineligible clients (e.g., with intellectual disability, memory impairment, acute psychological crisis, or inability to provide informed consent) is unknown. Although the participants represent a small proportion of the caseload, we consider it representative for the caseload, as the group was heterogeneous, encompassing a range of diagnoses and a variety of attitudes toward POD (positive, neutral, and critical).*

*We recognize that individuals who chose to participate may differ from those who declined; people less inclined to share their views might hold different perspectives, which could influence the findings. Individual differences in communication and familiarity with POD were not systematically accounted for, which may also have shaped the results. Moreover, the social network perspective - central to POD - was underrepresented, with only two interviews including network participants. Possible barriers included timing (during office hours), limited network involvement in the treatment, and client hesitancy. While more proactive support to involve network members might have increased participation, clients’ autonomy was prioritized.”*

In addition, the anonymized and aggregated findings were presented to the treatment team, who confirmed that the results were consistent with their broader clinical observations and offered no additional feedback. Together with the assessment of data saturation during analysis, this supports the credibility and transferability of the findings despite the small sample. We have revised the manuscript accordingly (see Strengths and Limitations, lines 460-464 in the Revised Manuscript without Track Changes):

*“Despite these limitations, we observed that data saturation was reached during analysis. In addition, the anonymized, aggregated findings were presented to the treatment team, who confirmed their consistency with broader clinical experience and offered no additional feedback. Together, these observations support the credibility and transferability of the findings despite the small sample, which should be interpreted as transferable insights rather than statistically generalizable results.”*

Finally, we expanded the *Conclusion and Future Research* section to emphasize the importance of recruiting a broader range of participants in future work (See Conclusion and future research, lines 472-473 in the Revised Manuscript without Track Changes):

“Future research should intentionally broaden the participant base to include clients - particularly those less inclined to participate - as well as a wider range of professionals and social network members, to deepen understanding of how mental health professionals can optimally support recovery.

**Reviewer #2:**

1. Authors provided a qualitative study to deepen the understanding of clients’ lived experiences with Peer-supported Open Dialogue to identify the specific behaviors, skills, and organizational elements they consider beneficial to their recovery.
   Although the topic is interesting, the manuscript’s readability is limited by the lack of tabulated data and illustrative figures. It is therefore recommended to enrich the text with tables and visual materials to improve clarity and accessibility, both in the presentation of the results and in the theoretical exposition of the POD model and its integration into the clinical workflow of MHCs.

Response: Thank you for this valuable suggestion. To improve readability and in line with the recommendations of the editor and other reviewer, we have substantially reduced the manuscript, roughly halving its length. We have also added a table in the Introduction summarizing the core OD principles (see Introduction, line 85 in the Revised Manuscript without Track Changes), as well as a summary table in the Results section to present the findings clearly and concisely (see Results, line 210 in the Revised Manuscript without Track Changes). To maintain the flow of the article, we propose moving Table 1 (line 85) to the appendix.

Regarding the suggestion to include a figure illustrating how POD is integrated into the clinical workflow of MHCs, we opted not to add a separate figure. We realize that this may not have been sufficiently clear in the original manuscript. In fact, the POD approach represents the full clinical workflow, encompassing the entire treatment process from initial contact to completion. Within this practice, additional evidence-based treatments, interventions, and support can be offered as needed. Rather than a figure, we have now explicitly described this workflow in the Methods section to clarify how POD structures the clinical process in practice (see Setting, lines 117-123 in the Revised Manuscript without Track Changes).

*“At the studied site, the POD team operates in accordance with the OD approach, as outlined in the Introduction and summarized in Table 1, with prior studies detailing its implementation and challenges [17,45]. The team integrates peer workers - professionals with lived experience - and embodies the ethos of “nothing about us, without us” [33–35]. POD entails a continuous, person-centered treatment pathway, encompassing the full clinical workflow from initial contact to completion. Network meetings form a central component, while additional treatments, interventions, and support are flexibly offered according to each client’s individual needs and preferences.”*

We hope that these revisions sufficiently address the reviewer’s concern and improve the clarity and accessibility of the manuscript.

6. PLOS authors have the option to publish the peer review history of their article ([what does this mean?](https://eur03.safelinks.protection.outlook.com/?url=https%3A%2F%2Ftrack.editorialmanager.com%2FCL0%2Fhttps%3A%252F%252Fjournals.plos.org%252Fmentalthealth%252Fs%252Feditorial-and-peer-review-process%2523loc-peer-review-history%2F1%2F010f01969267629d-b6428086-9953-4447-933b-a7ff0b659b29-000000%2FsnYI6xHNtIbgVfV93MNYNjJQ-QYIHikCLccG0GIUMME%3D209&data=05%7C02%7Ckarin.lorenz%40ggze.nl%7Cfe87afcc1c6747e2ea4a08dd9d2f4859%7C8e63ae1811a44bccac6a45f9a20e84ca%7C0%7C0%7C638839548595726362%7CUnknown%7CTWFpbGZsb3d8eyJFbXB0eU1hcGkiOnRydWUsIlYiOiIwLjAuMDAwMCIsIlAiOiJXaW4zMiIsIkFOIjoiTWFpbCIsIldUIjoyfQ%3D%3D%7C0%7C%7C%7C&sdata=XIVZVSdoQT88ZngJ4pkv5dVx8gYu585FlAYeqw5476U%3D&reserved=0)). If published, this will include your full peer review and any attached files.


**Do you want your identity to be public for this peer review?** If you choose “no”, your identity will remain anonymous but your review may still be made public.


For information about this choice, including consent withdrawal, please see our [Privacy Policy](https://eur03.safelinks.protection.outlook.com/?url=https%3A%2F%2Ftrack.editorialmanager.com%2FCL0%2Fhttps%3A%252F%252Fwww.plos.org%252Fprivacy-policy%2F1%2F010f01969267629d-b6428086-9953-4447-933b-a7ff0b659b29-000000%2FMz5aKIRHDncLJ_5M3gfQKJByyfwZ3x61nAVMXbFmOtE%3D209&data=05%7C02%7Ckarin.lorenz%40ggze.nl%7Cfe87afcc1c6747e2ea4a08dd9d2f4859%7C8e63ae1811a44bccac6a45f9a20e84ca%7C0%7C0%7C638839548595739668%7CUnknown%7CTWFpbGZsb3d8eyJFbXB0eU1hcGkiOnRydWUsIlYiOiIwLjAuMDAwMCIsIlAiOiJXaW4zMiIsIkFOIjoiTWFpbCIsIldUIjoyfQ%3D%3D%7C0%7C%7C%7C&sdata=u4eKre%2BMq0MTTxmx7HMwVXEycdvpLkXBm%2BCZH22GM3E%3D&reserved=0).

Reviewer #1: No

Reviewer #2: **Yes:** Michele Poletti
